# Body Composition Assessment and Mediterranean Diet Adherence in U12 Spanish Male Professional Soccer Players: Cross-Sectional Study

**DOI:** 10.3390/nu13114045

**Published:** 2021-11-12

**Authors:** Guillermo Santos-Sánchez, Ivan Cruz-Chamorro, José Luis Perza-Castillo, Néstor Vicente-Salar

**Affiliations:** 1 Departamento de Bioquímica Médica y Biología Molecular e Inmunología, Universidad de Sevilla, 41009 Seville, Spain; gsantos-ibis@us.es (G.S.-S.); icruz-ibis@us.es (I.C.-C.); 2 Instituto de Biomedicina de Sevilla, IBiS (Universidad de Sevilla, HUVR, Junta de Andalucía, CSIC), 41013 Seville, Spain; 3 Departamento de Ciencias de la Salud, Universitat Oberta de Catalunya, 08018 Barcelona, Spain; jperza@uoc.edu; 4 Biochemistry and Cell Therapy Unit, Institute of Bioengineering, University Miguel Hernandez, 03201 Elche, Spain; 5 Department of Applied Biology-Nutrition, Alicante Institute for Health and Biomedical Research (ISABIAL-FISABIO Foundation), University Miguel Hernandez, 03201 Elche, Spain

**Keywords:** KIDMED, nutritional habits, team sport, anthropometric parameters, young athletes

## Abstract

Soccer is the most practiced team sport in the world. Due to the importance of nutrition in soccer performance, controlling the body composition and dietary guidelines of players takes place starting from lower categories. The objective of this study was to evaluate body composition and adherence to the Mediterranean diet of U12 players from a professional soccer team and to identify their dietary weak points. Seventy-one U12 male soccer players participated in the study. Weight, height, percentiles, skinfolds, and body fat were measured by a certified anthropometrist following the procedures recommended by the International Society for the Advancement of Kinanthropometry. The Mediterranean diet adherence test (KIDMED) was the questionnaire used to evaluate eating habits. In addition, a comparison was made among field positions. The results showed percentiles and body fat percentages appropriate for their age. Furthermore, the average score on the KIDMED test showed that the players generally adhered well to the Mediterranean diet, although they should improve their consumption of fruits and vegetables, as well as avoid skipping breakfast. Moreover, goalkeepers and defenders had a higher percentile BMI and percentage of fat than midfielders and forwards. In addition, these players had lower KIDMED values than midfielders and forwards. Although U12 soccer players have an appropriate body composition and adherence to the Mediterranean diet, there are differences between the different field positions that should be assessed by coaches, doctors, and nutritionists/dietitians.

## 1. Introduction

Soccer is the most popular sport in the world, with approximately 265 million registered players [1]. It is an intermittent team sport characterized by large amounts of low-intensity actions interspersed with frequent bouts of high-intensity actions (acceleration and decelerations, rapid changes in directions, jumping, and landing tasks) [2]. In addition, players must be involved in several contact situations with opponents, in order to keep possession of or to win the ball [3]. During a match, soccer players can cover distances between ~5 and 7 km in U12 players and ~8 and 13 km in professional senior players [4,5]. For this reason, in addition to training, diet is a very important factor that influences sports performance and recovery [6].

Numerous associations, such as the American Association of Dietitians, the Dietitians of Canada, the American College of Sports Medicine of physical activity, the International Society of Sports Nutrition (ISSN) and the UEFA expert group position, underline the role of diet and how athletic performance and recovery from exercise are enhanced by optimal nutrition [7,8,9]. According to the recommendations of these organizations, the Mediterranean diet is an interesting option, not only because it ensures good health, but also because it can improve performance in some physical skills [10,11,12]. This diet is characterized by: high and varied consumption of fruits and vegetables; varied consumption of legumes and whole grains; extra virgin olive oil, nuts and seeds as the main source of fat; moderate consumption of fish; low consumption of red and processed meats; and moderate consumption of dairy products [13]. One study has demonstrated that the Mediterranean diet can improve endurance exercise performance in as little as 4 days [14]. For this reason, the study of adherence to this diet has attracted interest, with the KIDMED test being used in children and youths as the best tool to check it [15].

Soccer benefits from having one of the highest rates of participation among children and adolescents throughout the world. For some years, soccer clubs have begun to operate in subcategories, since, in this way, players will be trained as soccer players, receiving technical and tactical lessons, and the necessary standards to achieve success. In addition, working with lower categories can be very beneficial for soccer clubs, since players will become familiar with the club’s work philosophy, adapting easily to the demands of each category. In Spain, soccer teams of lower categories are classified according to the age of their players: pre-youngest (5–8 years), youngest (9–10 years), “alevines” (11–12 years), infants (13–14 years), cadets (15–16 years), youth (17–19 years) and seniors (>19 years) [16].

In recent years, professional clubs have been working to educate their youngest players that they have to start implementing a series of healthy habits that will be the foundation of their health, growth, sexual development, and daily performance. The development of a soccer player, in the lower categories, will depend on optimal nutrition, among other factors [17]. This fact is becoming more and more clear and there is increasing interest, among relatives of these young players, in providing them with routines and behaviors for hygiene and daily feeding. For this reason, many clubs have included a specific medical team (doctor, sports dietitians, physiotherapist, etc.) for lower categories, who work together in providing carefully designed nutrition education programs to parents and children, in addition to having control of the body composition of players [17].

Because it is a recent field of study, the data on nutritional and body composition characteristics through anthropometric measures of lower-level soccer players are scarce. There are enough anthropometric data in professional teams U13–U19 [18,19,20,21], but in lower categories, the anthropometric data are limited, especially when it comes to seeing differences between field positions [21]. In addition, to date, there are no data about adherence to the Mediterranean diet in U12 professional soccer players. Therefore, the purposes of this study were (1) to evaluate the body composition of U12 players; (2) to assess the nutritional composition and adherence to the Mediterranean diet of U12 soccer players using the KIDMED test; (3) to analyze variations in body composition and adherence to the Mediterranean diet among field positions; and (4) to evaluate the ability of the KIDMED test to predict variations in body composition.

## 2. Materials and Methods

### 2.1. Participants

**Figure 1:**
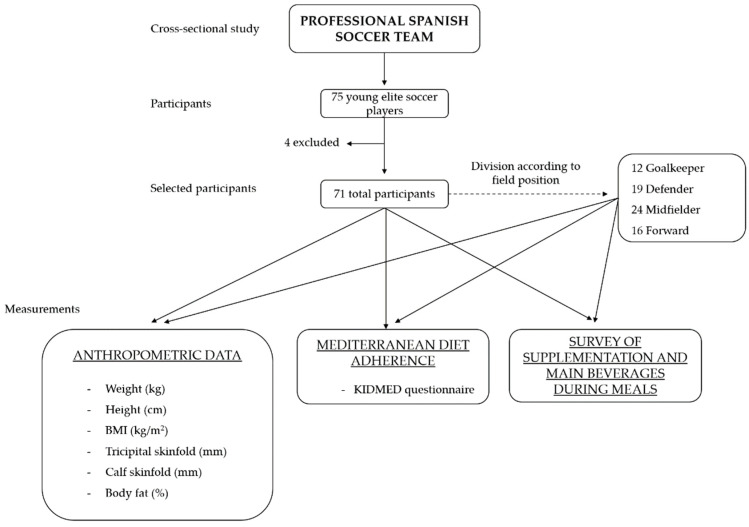
Schematic representation of the study. Seventy-five young male soccer players of the professional Spanish soccer team were recruited. Finally, 4 players were excluded for not having a fixed position on the field. Of the remainder, 12 were goalkeepers, 19 defenders, 24 midfielders, and 16 forwards. Body composition, adherence to the Mediterranean diet, main drinks, and supplements were collected. The analysis was carried out in the global team and by field position.

Seventy-five male soccer players from the low categories of a professional club were collected to participate in the study. Specifically, these belonged to the youngest (8–10 years) and “alevines” (10–12 years) teams. The study was carried out during the regular competition season (September–June). Parents or legal guardians of the participants were informed about the study objective and gave their written consent to participate. Anonymity was preserved for all participants. The inclusion criteria used were: (1) aged between 8 and 12 years old, and (2) well-defined field position. Participants were excluded if (1) they were female, (2) parents or legal guardians did not sign the informed consent or (3) participants were currently under any medical treatment. Finally, 71 soccer players were considered in this study, since 4 were excluded for not having a fixed position on the field. The study design is schematized in Figure 1.

### 2.2. Data Collection

The data were collected over a week. The participants were accompanied by a family member who participated in answering the questionnaires. All data collection was carried out by a dietitian. The date of birth, the team he belonged to, and his position on the field (goalkeeper, defender, midfielder, forward) were required.

#### 2.2.1. Anthropometric Measurements

Measurements were made by a certified anthropometrist in accordance with guidelines outlined by the International Society for the Advancement of Kinanthropometry (ISAK), with an individual technical error of measurement (TEM) of 0.76% for skinfolds and 0.12% for the remaining parameters, both in the range of ISAK accreditation (<7.5% for skinfolds and <1.5% for the rest of measurements).

Body composition parameters, including weight (kg), height (cm), body mass index (BMI) (kg/m^2^), body skinfolds (mm) and body fat (kg and %), were measured during the mid-season competition. Height was determined using a stadiometer (Seca^©^ 213 stadiometer, Seca, Hamburg, Germany) with the participant’s head held at the position of the Frankfort horizontal plane. Weight was measured with bioelectric impedance analysis (Tanita BC-418, Tokyo, Japan). BMI was then calculated by dividing body mass by height squared. In addition, the triceps and medial calf skinfolds were evaluated using a plicometer (Smartmet, Crymych, United Kingdom). Each measurement was taken two times, in accordance with the recommendations of the ISAK. The body fat percentage was estimated using the equation proposed by Slaughter [22]: (body fat percentage = 0.735 (triceps skinfold + calf skinfold) + 1.0); this is specific to children and adolescents. Lastly, percentiles of weight, height, and BMI were calculated according to the Centers for Disease Control and Prevention (CDC) [23].

#### 2.2.2. Evaluation of the Mediterranean Diet Quality Index

To evaluate adherence to the Mediterranean diet, the KIDMED test was used. The KIDMED test is a 16-item yes/no questionnaire that is a valid tool to evaluate the quality of eating habits of children and adolescents [24]. Items are shown in Table 1. If the participant answers affirmatively to the items with a positive connotation (1, 2, 3, 4, 5, 7, 8, 9, 10, 11, 13, 15), a +1 was added to their score. However, for positive answers to questions with a negative connotation (6, 12, 14, 16), the participant obtains a −1 to the score. The final result of the KIDMED test is the sum of all the items. The assessment of the test is carried out through the following classifications: very poor-quality diet (Low Adherence): total score ≤3; need to improve dietary pattern to adapt it to the Mediterranean model (Average Adherence): total score between 4 and 7; optimal Mediterranean diet (High Adherence): total score ≥8.

#### 2.2.3. Supplementation and Beverages Survey

Participants were also asked what kind of beverages usually accompany meals and if they take supplements frequently. The self-compiled survey was carried out in the same way as the KIDMED questionnaire.

### 2.3. Statistical Analysis

Data are expressed as mean ± standard deviation (SD) and were analyzed by a non-parametric binomial test or by Mann–Whitney U test with IBM^®^ SPSS^®^ Statistic software v.26 (IBM, Armonk, NY, USA). Two groups were conducted to evaluate the differences between back and front field positions (Group 1, goalkeepers and defenders; Group 2, midfielders and forwards). Correlations were analyzed by the non-parametric Spearman’s correlation. *p* values *p* ≤ 0.05 were considered statistically significant.

## 3. Results

### 3.1. Participant Characteristics

Seventy-one male soccer players were considered in this study, and they were grouped into the age categories of Spanish soccer. The physical characteristics of the players are reported in Table 2. Significant differences were observed in age, weight, height, BMI, body fat and body fat percentage between both age groups (Table 2).

### 3.2. Anthropometric Characteristics and Body Composition of Different Field Positions

Of the 71 players who completed the study, 12 were goalkeepers, 19 were defenders, 24 were midfielders, and 16 were forwards. Thus, there were significant differences (*p* < 0.05) in the parameters analyzed between the different field positions except for age, height, and height percentile (Table 3). The goalkeepers presented the greatest differences compared to the rest of the team positions. In fact, they showed a significantly higher weight (37.53 ± 4.91 kg) and weight percentile (74.26 ± 14.24%) than defenders (33.88 ± 3.69 kg, *p* = 0.022; 57.75 ± 16.67%, *p* = 0.007), midfielders (33.79 ± 5.16 kg, *p* = 0.05; 55.81 ± 22.27%, *p* = 0.006), and forwards (32.86 ± 5.57 kg, *p* = 0.027; 52.94 ± 24.33%, *p* = 0.018).

It is observed how the weight percentile decreases from the defender positions to the front (Goalkeepers > Defenders > Midfielders > Forwards). This trend is also clearly reflected in other parameters such as BMI, BMI percentile, body skinfolds, and the percentage and weight of the body fat.

Focusing on BMI, a significant difference between BMI and BMI percentile in regard to goalkeepers (18.13 ± 2.08 kg/cm^2^ and 64.69 ± 25.15%) and forwards (16.65 ± 1.16 kg/cm^2^ and 44.73 ± 23.41%) (*p* = 0.046 and 0.041, respectively) was observed. In addition, the present study showed that the triceps skinfold is significantly higher in goalkeepers (11.44 ± 3.88 mm) in comparison to forwards (8.05 ± 2.05 mm) (*p* = 0.031). Similarly, goalkeepers presented a significantly higher calf skinfold (10.45 ± 3.15 mm) in comparison to midfielders (8.19 ± 2.99 mm) (*p* = 0.011) and forwards (7.55 ± 2.69 mm) (*p* = 0.004). These data were reflected in the body fat and body fat percentage, which was significantly higher in goalkeepers (6.51 ± 2.28 kg and 17.09 ± 4.86%, respectively) than midfielders (4.97 ± 2.19 kg, *p* = 0.032; 13.91 ± 3.88%, *p* = 0.049) and forwards (4.28 ± 1.66 kg, *p* = 0.007; 12.47 ± 3.26%, *p* = 0.013).

In addition, the anthropometric parameters by field positions were analyzed in terms of age groups (Supplementary Tables S1 and S2). No significant differences in body composition were observed between the field positions in the 8–10 years group. However, in the 10–12 years group, goalkeepers showed higher BMI values (18.93 ± 1.91 kg/m^2^) than forwards (16.88 ± 1.36 kg/m^2^) (*p* = 0.025), as well as a higher BMI percentile (69.10 ± 24.90) than midfielders (49.93 ± 18.30) and forwards (45.33 ± 20.88) (*p* = 0.039 and 0.042, respectively). Furthermore, and focusing on body fat, goalkeepers presented a higher calf skinfold (11.66 ± 3.34 mm) and body fat percentage (18.48 ± 5.06%) in comparison to midfielders (8.44 ± 3.42 mm, *p* = 0.048; 14.43 ± 4.52%, *p* = 0.048) and forwards (7.90 ± 3.27 mm, *p* = 0.037; 13.07 ± 3.68%, *p* = 0.031).

### 3.3. Adherence to the Mediterranean Diet

To identify the adherence to the Mediterranean diet, the KIDMED test was used. In general, the players reached an average KIDMED score of 7.83 ± 2.03, showing that the players have high adherence to the Mediterranean diet (optimal Mediterranean diet with a total score ≥ 8). As shown in Table 4, 81.69% of the players consume at least one piece of fruit or natural juice per day (Item 1) (*p* < 0.001); however, only half (50.70%) consumed a second piece of fruit (Item 2) (*p* < 0.001). As for vegetables, only 54.93% consume them daily (Item 3) (*p* = 0.477), while 80.28% do not consume a second vegetable a day (Item 4) (*p* < 0.001). On the other hand, 70.42% of the players have a good consumption of fish (Item 5) (*p* = 0.001), 90.14% of complex carbohydrates (Item 8) (*p* < 0.001), and 98.59% of legumes (Item 7) (*p* < 0.001). As for breakfast, 85.92% of the players have cereal or derivatives for breakfast (Item 9) (*p* < 0.001) and 91.55% accompany it with dairy (Item 13) (*p* < 0.001). However, at least 21.13% of young soccer players skip breakfast (Item 12). Focusing on the consumption of unsaturated oils (Item 11), it stands out that extra-virgin olive oil is used for cooking for 100% of players (*p* < 0.001). However, 71.83% do not consume nuts more than 2 or 3 times a week (Item 10) (*p* < 0.001). Finally, evaluation of the consumption of fast food and pastries showed how almost one-quarter of players (23.94%) include commercially produced pastries in their breakfast (Item 14) (*p* < 0.001), and 18.31% consume sweets several times a day (Item 16) (*p* < 0.001), although 84.51% do not frequently visit fast-food restaurants (Item 6) (*p* < 0.001).

No differences in the total KIDMED score were observed between the age groups (Supplementary Table S3).

On the other hand, there were no significant correlations between the KIDMED test and the anthropometric parameters (weight, BMI, and body fat percentage) (Supplementary Table S4).

### 3.4. Body Composition and Nutritional Behaviors

**Figure 2:**
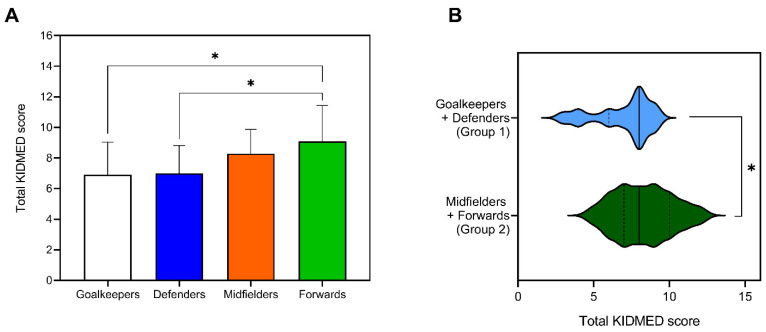
Adherence to the Mediterranean Diet assessed by the KIDMED index. (**A**) KIDMED test total score in the different playing positions; (**B**) KIDMED test total score when players are divided by field position in Group 1 (Goalkeepers and Defenders) and Group 2 (Midfielders and Forwards). *, *p* ≤ 0.05.

The KIDMED test was evaluated in each of the positions on the field in order to find a correlation in feeding with the differences in the anthropometric characteristics observed. There were significant differences in the KIDMED score regarding different field positions, as shown in Figure 2A. The worst scores in the KIDMED test were obtained in the players who played as goalkeepers (6.90 ± 2.13) and defenders (7.00 ± 1.82), while the highest scores were obtained in midfielders (8.29 ± 1.57) and forwards (8.85 ± 2.41). In fact, a significant difference in the KIDMED total scores was observed when comparing the back positions of the field (Group 1) (Goalkeeper + Defender) with the front positions (Group 2) (Midfielder + Forward) (*p* = 0.024) (Figure 2B).

The players present in Group 1 reported the highest percentages of body fat and BMI percentile, which could be associated with bad eating habits. Once the analysis of the 16 items that make up the KIDMED test (Table S2) was performed, it was observed that although both groups have a similar consumption of a piece of fruit daily (80.6 vs. 82.5%), only 41.94% of the players in Group 1 consumed a second piece of fruit compared to 57.5% of Group 2. On the other hand, 45.16% of the players in Group 1 eat one vegetable daily, and only 12.9% a second vegetable. However, of Group 2, 62.5% consume one vegetable a day (17.4% more than Group 1), while 25% consume at least a second (twice than Group 1).

Regarding breakfast, 93.5% of the subjects of Group 1 claimed to include cereal or derivatives for breakfast, as opposed to 80% of Group 2 (13.5% less than Group 1). In addition, 32.6% of the subjects of Group 1 responded affirmatively to consuming a breakfast of commercially produced pastries, against 17.5% of Group 2. Finally, it is noteworthy that 25.8% of the players belonging to Group 1 confirmed consuming sweets several times a day, a percentage that is twice that of those who consume sweets in Group 2 (12.5%).

### 3.5. Supplements and Beverage Consumption

**Figure 3:**
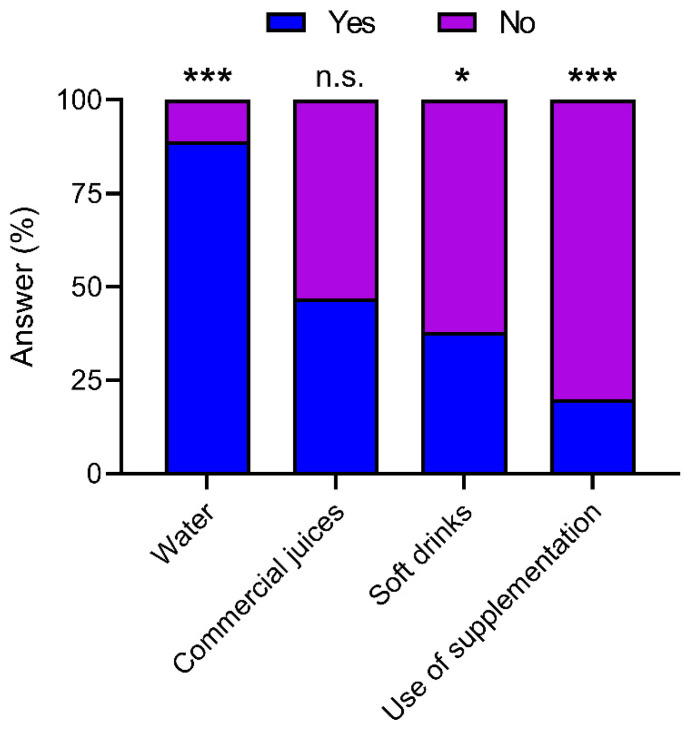
Main beverage in meals and use of supplements for the total sample. *, *p* ≤ 0.05; ***, *p* ≤ 0.001; n.s., not significant.

As shown Figure 3, when players were asked about their main beverages at meals, the most reported as being consumed were water (89.2%), commercial juices (47.3%), and soft drinks (28%). In addition, 20.3% of young soccer players consume dietary supplements daily (i.e., carbohydrate–electrolyte drinks).

When dividing by field position (Table S3), no significant differences were observed between the two groups in terms of consumption of water, commercial juices, and soft drinks (*p* > 0.05). However, 25% of the players that comprised Group 1 claimed to consume dietary supplements, which was significantly higher than 6.7% of Group 2 (*p* = 0.038).

## 4. Discussion

Millions of children around the world are involved in soccer [25,26]. Although most children play soccer recreationally, the number of them who are federated from a very young age by joining a professional club has increased notably [25,26]. It is well known that practicing soccer has beneficial effects on numerous health parameters of children and adolescents [27,28,29]. In this sense, a recent study has demonstrated that U12 male soccer players had better exercise capacity, lower resting heart rate, and higher muscle mass than children that do not play soccer [30,31]. Due to soccer having the highest media coverage among sports and being the one that generates the most income in the world [32], coaches are persistently searching for the best methods to identify and develop talented young soccer players. The supposed role of the lower categories in soccer is to act as centers for the selection and development of prospective young soccer players [33]. In this sense, from the lowest category of the club (U8), the club’s staff begins to focus on the diet and body composition of its players [34], to attain their best performance. Thus, the main purpose of this investigation was to assess the body composition and adherence to the Mediterranean diet of top-level U12 soccer players. Controlling these parameters could help to optimize the sports performance of future professional players. The present study also analyzed the differences among playing positions (goalkeepers, defenders, midfielders, and forwards).

Focusing on body composition, the U12 soccer players presented mean values of weight, height, and BMI appropriate for their age range. In fact, players would be around the 50th percentile, which is considered normal weight by the National Center for Health Statistics [23]. In addition, the body fat percentage was 14.44 ± 0.55%, which is also considered a normal body fat level in childhood [35]. It is important to highlight the limitation of the use of the Slaughter equation to calculate body fat, since it has been shown to underestimate body fat percentage [36].

Previous research has considered the importance of the control of these values in soccer. Nikolaidis demonstrated that an elevated BMI and body fat percentage are associated with decreased physical fitness in young soccer players [37]. These results (both BMI and body fat percentage) are well below the values of the young Spanish population. In fact, it is reported that the body fat percentage in Spanish children between 8 and 11 years is 24% [38,39]. Furthermore, the results of the present study are in accordance with Campa et al., which showed that the BMI values of U10, U11, and U12 Italian male professional soccer players are 16.9 ± 1.4 kg/m^2^, 17.1 ± 1.5 kg/m^2^, and 18.7 ± 1.7 kg/m^2^, while the body fat percentage values are 12.8 ± 2.2%, 14.0 ± 3.2%, and 15.9 ± 3.9%, respectively [40]. The present study provides robust data (*n* = 71) about the body composition of U12 male professional soccer players. Additionally, in our study, players between 8 and 10 years old were observed to have a lower body fat percentage (13.43 ± 4.91%) than the 10–12 years group (15.11 ± 4.58%).

Other studies have demonstrated that body fat percentage has a significant and negative correlation with age [18,40]. Players from professional U15, U17, and U19 soccer teams had 12.9 ± 6.8%, 10.0 ± 3.6%, and 10.4 ± 3.9% body fat, respectively [18], a fact that also occurs in other sports (e.g., basketball) [41]. This fact would explain why our U12 players present a higher body fat percentage, perhaps because they had not fully matured to the point where adipose mass decreases and muscle mass increases [18]. However, when our results were divided by age group (8–10 and 10–12 years), this trend was not observed. These results are in agreement with Moreno et al., which showed how younger soccer players (9–14 years) do not tend to this correlation, due to development and maturity processes [42].

In addition, the present study evaluated whether there were differences in body composition between the different field positions. The results showed how the goalkeepers had a higher height percentile than forwards in the 8–10 years group but not in the 11–12 years group. There was no significant difference between the parameters analyzed in the 8–10 years group, but a trend was observed in the increase in fat mass from the most defensive positions to the most offensive ones. More volunteers in future studies, especially goalkeepers, would be necessary to clarify this fact. Moreover, the 10–12 years group showed how the goalkeepers had the highest BMI percentile and body fat percentage values, while forwards showed the lowest ones. In addition, it is observed that there is a decrease in the levels of BMI percentile and body fat percentage as the position of the player moves away from the goalkeeper (goalkeepers > defenders > midfielders > forwards). These results are in accordance with what was observed in higher categories (U14–U20) [21,43]; however, thanks to this study, it can be concluded that the anthropometric differences between field positions already exist from the lowest categories (U12). This outcome could be attributed to the fact that goalkeepers are subject to a lower metabolic rate than other players during games and training [18,44]. In addition, the tactical maneuvers performed by forwards result in high levels of energy expenditure [44]. For this reason, the nutritional needs should be adapted to the field position from the moment the players begin to play in a professional team, regardless of age.

Regarding the assessment of adherence to the Mediterranean diet, the present study is the first that evaluates diet quality using the KIDMED test in U12 male professional soccer players. In this study, the young soccer players showed a mean KIDMED score of 7.83 ± 2.03 (with no differences between age groups), which is considered as Optimal Mediterranean Diet (High Adherence) [15,45]. However, some items must be analyzed in depth. The consumption of fruits and vegetables continues to be the point needing to be solved [46]. In fact, other young athletes such as kayakers have demonstrated the same problem with the consumption of fruits and vegetables [47]. This is an important question that needs to be improved, since many studies have demonstrated a relationship between low consumption of fruits and vegetables and low physical activity [48,49]. On the other hand, the present study has shown that at least 20% of the players skip breakfast and that 21.13% of the players eat commercially produced pastries for breakfast. In this sense, the bibliography has shown that skipping breakfast or consuming an unhealthy breakfast affects athletic performance [50,51,52]. Philippou et al. showed that a half-day nutrition education session was enough to improve adherence to the Mediterranean diet by 2 points in the KIDMED test of a group of professional swimmers [53].

Moreover, the score obtained in the KIDMED test by field position was evaluated. The results show that, although all groups have a high score, it decreases as the player’s position moved away from the goalkeeper (goalkeepers > defenders > midfielders > forwards), in the same way that we have observed in some anthropometric parameters (BMI, BMI percentile, weight percentile, and body fat percentage). In fact, the relationship between these anthropometric parameters and a worse score in the quality of the Mediterranean dietary pattern has been evidenced, a circumstance that does not require a detailed analysis because it is widely documented [54]. In our study, it seems that it is the consumption of at least a second piece of fruit, the consumption of more vegetables, and avoiding an unhealthy breakfast are responsible for the differences observed between groups.

Although we have found significant differences in body composition and KIDMED score by field position, no significant correlations were observed between these parameters. This result is in accordance with the study by Martinez et al. [55]. Specifically, they also did not observe significant correlations between the KIDMED score and body composition parameters in senior and junior beach handball players [55]. We conclude that, although the KIDMED test is a good identifier of bad eating habits, it is not a good predictor of variations in body composition in U12 male soccer players. Future studies in other categories, and including female soccer players, are needed.

On the other hand, 47.5% of players include commercial juice and 37.8% soft drinks among the main drinks of their meals. Certain studies have demonstrated the strong relationship between the consumption of these kinds of drinks, containing free-sugars, and the prevalence of obesity in the child population [56]. For this reason, the family and the technical staff team should encourage the consumption of water during main meals and during practices, since scientific evidence has shown that many soccer players reach significant dehydration during games, compound by initial hypohydration [57,58]. In addition, 25% of forward/midfielder players stated that they consume dietary supplements, a fact that is not necessary for this age group as long as they have an adequate diet [59].

This study presents certain limitations that must be explained. The KIDMED test served to evaluate the quality of the players’ diet, but not the quantity. In addition, due to the nature of the study, the data collection using the KIDMED questionnaire could lead to an error in the reports and memory bias. It would be interesting to be able to make 7-day records to be able to assess both quality and quantity. Another limitation is the lack of data regarding the maturity level and the sport performance of the young soccer players. It could provide better information about differences between age categories and even if the position on the field is chosen because of their maturity. However, this last limitation is frequent in other similar studies [43].

In future studies, it would be interesting to evaluate the relationship among adherence to the Mediterranean diet, taking into account quantities, body composition, and the physical performance of soccer players.

## 5. Conclusions

This study robustly (*n* = 71) shows that (1) U12 Spanish male professional soccer players have an appropriate body composition. In addition, the present study, which is pioneer in the use of a validated test to evaluate the diet quality of young soccer players, demonstrates that (2) the young players have a suitable adherence to the Mediterranean diet, (3) although some nutritional habits need to be improved. In addition, this study shows (4) the importance of adopting nutritional recommendations from very early ages as the differences in body composition in terms of field position showed. Finally, the results of the KIDMED test indicate that, although it should not be used as a predictor of variations in the body composition of U12 male soccer players, (5) this test is suitable for identifying the key nutritional points that should be worked on from the point of view of nutritional education.

## Figures and Tables

**Table 1:** Mediterranean Diet Quality Index (KIDMED).

KIDMED Test	Scoring
1. Consume natural fruit juice or fruit juice every day	+1
2. Have a second fruit every day	+1
3. Eat fresh or cooked vegetables once a day	+1
4. Eat fresh or cooked vegetables more than once a day	+1
5. Consume fish at least 2–3 times a week	+1
6. Go once or more times a week to a fast-food restaurant (burger joint, pizzeria, etc.)	−1
7. Eat legumes more than once a week (chickpeas, beans, lentils, peas, etc.)	+1
8. Consume pasta, rice, bread, and potato almost every day (5 or more per week)	+1
9. Have cereals or grains (bread, etc.) for breakfast	+1
10. Consume nuts at least 2 or 3 times a week (walnuts, hazelnuts, almonds)	+1
11. Use olive oil at home	+1
12. Skip breakfast	−1
13. Have a dairy product for breakfast (yogurt, milk, etc.)	+1
14. Have commercially baked goods or pastries for breakfast	−1
15. Consume two yogurts and/or some cheese daily	+1
16. Consume sweets and candy several times every day	−1

**Table 2:** Anthropometric parameters.

	Mean ± SD		
Anthropometric Parameters	Total (*n* = 71)	8–10 Years (*n* = 26)	10–12 Years (*n* = 45)
Age (years)	10.19 ± 1.17	8.95 ± 0.63	11.01 ± 0.53 ***
Weight (kg)	34.51 ± 5.43	30.36 ± 3.98	37.27 ± 4.44 ***
Height (cm)	141.42 ± 8.37	135.15 ± 6.25	145.60 ± 6.88 ***
BMI (kg/m^2^)	17.17 ± 1.54	16.59 ± 1.54	17.55 ± 1.42 **
Weight percentile	58.36 ± 21.95	60.39 ± 24.89	57.02 ± 19.94
Height percentile	59.72 ± 27.41	59.20 ± 27.78	60.07 ± 27.48
BMI percentile	54.43 ± 22.21	55.07 ± 24.78	54.01 ± 20.60
Triceps skinfold (mm)	9.55 ± 3.23	8.81 ± 3.18	10.04 ± 3.20
Calf skinfold (mm)	8.74 ± 3.56	8.11 ± 3.77	9.16 ± 3.40
Body fat (Slaughter equation) (%)	14.44 ± 4.75	13.43 ± 4.91	15.11 ± 4.58 *
Body fat (kg)	5.11 ± 2.28	4.19 ± 2.13	5.73 ± 2.18 ***

*, *p* ≤ 0.05; **, *p* ≤ 0.01; *p* ≤ 0.001 compared to the 8–10 years group. SD, standard deviation; BMI, body mass index.

**Table 3:** Anthropometric parameters by field position for total sample.

Anthropometric Parameters	Goalkeeper (*n* = 12)	Defender (*n* = 19)	Midfielder (*n* = 24)	Forward (*n* = 16)
Age (years)	10.19 ± 1.32	10.16 ± 0.97	10.34 ± 1.34	10.21 ± 1.13
Weight (kg)	37.53 ± 4.91	33.88 ± 3.69 **	33.79 ± 5.16 **	32.86 ± 5.57 *
Height (cm)	143.92 ± 7.04	139.88 ± 6.39	142.27 ± 8.30	141.57 ± 11.31
BMI (kg/m^2^)	18.13 ± 2.08	17.28 ± 1.02	17.01 ± 1.21	16.65 ± 1.16 *
Weight percentile	74.26 ± 14.24	57.75 ± 16.67 **	55.81 ± 22.27 **	52.94 ± 24.33 *
Height percentile	71.18 ± 26.81	54.31 ± 24.80	60.88 ± 25.46	57.46 ± 32.80
BMI percentile	64.69 ± 25.15	58.52 ± 16.01	51.46 ± 19.51	44.73 ± 23.41 *
Triceps skinfold (mm)	11.44 ± 3.88	9.37 ± 3.19	9.37 ± 2.57	8.05 ± 2.05 *
Calf skinfold (mm)	10.45 ± 3.15	8.65 ± 3.11	8.19 ± 2.99 *	7.55 ± 2.69 **
Body fat (Slaughter equation) (%)	17.09 ± 4.86	14.25 ± 4.34	13.91 ± 3.88 *	12.47 ± 3.26 *
Body fat (kg)	6.51 ± 2.28	4.87 ± 1.77	4.97 ± 2.19 *	4.28 ± 1.66 **

Data are represented as mean ± standard deviation (SD). *, *p* ≤ 0.05; **, *p* ≤ 0.01 compared to the goalkeeper group. BMI, body mass index.

**Table 4:** Mediterranean diet quality index statistics for total sample.

Total KIDMED Score		7.83 ± 2.03	
ITEMS	Yes (%)	No (%)	*p*-Value
1	81.69	18.31	<0.001
2	50.70	49.30	1.000
3	54.93	45.07	0.477
4	19.72	80.28	<0.001
5	70.42	29.58	0.001
6	15.49	84.51	<0.001
7	98.59	1.41	<0.001
8	90.14	9.86	<0.001
9	85.92	14.08	<0.001
10	28.17	71.83	<0.001
11	100.00	0.00	<0.001
12	21.13	78.87	<0.001
13	91.55	8.45	<0.001
14	23.94	76.06	<0.001
15	70.42	29.58	0.001
16	18.31	81.69	<0.001

## Data Availability

Data are included within the article and Supplementary Materials.

## Supplementary Materials

The following are available online at https://www.mdpi.com/article/10.3390/nu13114045/s1, Table S1. Anthropometric parameters by field position in the 8–10 years group. Table S2. Anthropometric parameters by field position in the 10–12 years group. Table S3. Mediterranean diet quality index in the different groups. Table S4. Non-parametric Spearman’s correlations. Table S5. Details of the responses for each item of the KIDMED test by groups. Table S6. Main beverages consume and use of supplements in the total sample.
