# Supplementary material for: Body Composition Assessment and Mediterranean Diet Adherence in U12 Spanish Male Professional Soccer Players: Cross-Sectional Study
Source: Nutrients. 2021 Nov 12;13(11):4045. doi: 10.3390/nu13114045 (PMC8623059; doi:10.3390/nu13114045)
Supplement: Supplementary file 1 [file nutrients-13-04045-s001.zip › nutrients-1412987-supplementary.pdf]

nutrients MDPI

Supplemental material

Body Composition Assessment and Mediterranean Diet Adherence in U12 Spanish-male Professional Soccer Players: Cross-Sectional Study

Guillermo Santos-Sánchez†, Ivan Cruz-Chamorro†, José Luis Perza-Castillo, Néstor Vicente-Salar

Table S1. Anthropometric parameters by field position in the 8-10 years group.

Antropometric parameters	Goalkeeper (*n*=4)	Defender (*n*=8)	Midfielder (*n*=8)	Forward (*n*=6)
Age (years)	8.57 ± 0.38	9.22 ± 0.67	8.62 ± 0.49	9.05 ± 0.81
Weight (kg)	32.20 ± 2.03	31.86 ± 3.08	29.21 ± 2.90	29.33 ± 4.67
Height (cm)	139.25 ± 5.51	137.03 ± 6.53	133.74 ± 5.29	131.57 ± 6.90
BMI (kg/m^2)	16.70 ± 2.16	16.95 ± 0.86	16.31 ± 1.10	16.26 ± 1.44
Weight percentile	79.29 ± 14.11	65.47 ± 17.59	62.91 ± 16.72	44.11 ± 32.90
Height percentile	81.80 ± 20.28	61.62 ± 26.87	64.10 ± 20.49	38.38 ± 31.45*
BMI percentile	54.99 ± 29.80	61.25 ± 14.76	54.51 ± 22.76	49.50 ± 31.01
Triceps skinfold (mm)	9.10 ± 3.08	8.71 ± 3.24	8.45 ± 1.35	7.27 ± 1.97
Calf skinfold (mm)	8.45 ± 2.32	7.25 ± 2.64	7.69 ± 1.95	6.97 ± 1.35
Body fat (Slaughter equation) (%)	13.90 ± 3.79	12.73 ± 4.14	12.86 ± 1.95	11.46 ± 2.35
Body fat (kg)	4.53 ± 1.57	4.04 ± 1.30	3.79 ± 0.87	3.32 ± 1.13

Data are represented as mean ± standard deviation (SD). *, $p \leq 0.05$ compared to the goalkeeper group. BMI, body mass index.

nutrients

MDPI

Table S2. Anthropometric parameters by field position in the 10-12 years group.

Antropometric parameters	Goalkeeper (*n*=7)	Defender (*n*=11)	Midfielder (*n*=16)	Forward (*n*=10)
Age (years)	11.13 ± 0.59	10.84 ± 0.40	11.20 ± 0.51	10.91 ± 0.58
Weight (kg)	40.14 ± 3.71	35.35 ± 3.50**	37.43 ± 4.85	36.80 ± 4.68
Height (cm)	145.86 ± 7.25	141.96 ± 5.69	146.54 ± 5.86	147.58 ± 8.94
BMI (kg/m^2)	18.93 ± 1.91	17.52 ± 1.11	17.35 ± 1.14	16.88 ± 1.36*
Weight percentile	70.09 ± 14.87	52.13 ± 14.39*	52.27 ± 24.28	58.23 ± 17.39
Height percentile	62.46 ± 29.85	48.98 ± 22.97	59.26 ± 28.10	68.93 ± 29.22
BMI percentile	69.10 ± 24.90	56.52 ± 17.28	49.93 ± 18.30*	45.33 ± 20.88*
Triceps skinfold (mm)	12.13 ± 3.89	9.86 ± 3.21	9.83 ± 2.93	8.52 ± 2.04
Calf skinfold (mm)	11.66 ± 3.34	9.67 ± 3.14	8.44 ± 3.42**	7.90 ± 3.27**
Body fat (Slaughter equation) (%)	18.48 ± 5.06	15.35 ± 4.32	14.43 ± 4.52*	13.07 ± 3.68*
Body fat (kg)	7.40 ± 2.08	5.48 ± 1.87*	5.56 ± 2.43*	4.86 ± 1.71**

Data are represented as mean ± standard deviation (SD). *, $p \leq 0.05$; **, $p \leq 0.01$ compared to the goalkeeper group. BMI, body mass index.

nutrients MDPI

Table S3. Mediterranean diet quality index in the different groups

	8-10 years (*n* = 26)	11-12 years (*n* = 45)	p-value	Total (*n* = 71)
**KIDMED score**	7.50 ± 2.06	8.15 ± 1.93	0.210	7.83 ± 2.03

Mean of score values ± standard deviation obtained from the Mediterranean diet adherence test (KIDMED). p-value calculated between the 8-10 and 11-12 years groups.

nutrients MDPI

Table S4. Non-parametric Spearman's correlations

	**Spearman's r**	**p-value**
**Weight (kg)**	0.2042	0.103
**BMI (kg/m^2)**	-0,1343	0.279
**Body Fat (%)**	-0,1902	0.132

Non-parametric Spearman's correlations between KIDMED score and body composition parameters. BMI, body mass index.

Table S5. Details of the responses for each item of the KIDMED test by groups.

ITEMS	Group 1 Goalkeeper + Defender (n=31)		Group 2 Midfielder + Forward (n=40)	
	Yes (%)	No (%)	Yes (%)	No (%)
1	80,65	19,35	82,50	17,50
2	41,94	58,06	57,50	42,50
3	45,16	54,84	62,50	37,50
4	12,90	87,10	25,00	75,00
5	74,19	25,81	67,50	32,50
6	16,13	83,87	15,00	85,00
7	96,77	3,23	100,00	0,00
8	93,55	6,45	87,50	12,50
9	93,55	6,45	80,00	20,00
10	22,58	77,42	32,50	67,50
11	100,00	0,00	100,00	0,00
12	25,81	74,19	17,50	82,50
13	93,55	6,45	90,00	10,00
14	32,26	67,74	17,50	82,50
15	67,74	32,26	72,50	27,50
16	25,81	74,19	12,50	87,50

nutrients MDPI

Table S6. Main beverages consume and use of supplements by groups

	Group 1 Goalkeeper + Defender (n=30)		Group 2 Midfielder + Forward (n=40)	
	**Yes (%)**	**No (%)**	**Yes (%)**	**No (%)**
Water	28 (93,3%)	2 (6,7%)	35 (87,5%)	5 (12,5%)
Commercial juices	14 (46,7%)	16 (53,3%)	19 (47,5%)	21 (52,5%)
Soft drinks	10 (33,3%)	20 (66,7%)	15 (37,5%)	25 (62,5%)
Use of Supplements	2 (6,7%)*	28 (93,3%)	10 (25%)*	30 (75%)

* Significantly different between groups at p = 0.038.
